# Clinical Features and Prognosis Analysis of Hormone Receptor-Positive, HER2-Negative Breast Cancer with Differential Expression Levels of Estrogen and Progesterone Receptors: A 10-Year Retrospective Study

**DOI:** 10.1155/2022/5469163

**Published:** 2022-11-29

**Authors:** Jin Liu, Mingyu Gan, Zijing Lin, Qin Deng, Juan Deng, Bin Zeng, Yanling Shi, Jia Ming

**Affiliations:** ^1^Department of Breast and Thyroid Surgery, The Second Affiliated Hospital of Chongqing Medical University, No. 74, Linjiang Road, Yuzhong District, Chongqing 400010, China; ^2^Department of Breast and Thyroid Surgery, The First People's Hospital of Yibin, No. 65, Wenxing Street, Cuiping District, Yibin 644000, China; ^3^Department of Biochemistry and Molecular Biology, School of Basic Medical Science, Shanxi Medical University, Taiyuan 030001, China

## Abstract

**Background:**

Estrogen and progesterone receptor status can predict breast cancer patient prognosis and treatment sensitivity, but research on low ER and PR levels and expression balance remains limited.

**Methods:**

From January 2010 to October 2016, 283 ER+/PR+/HER2-breast cancer patients who met the inclusion criteria were enrolled and divided into the H group (ER > 10%, *N* = 261) and the *L* group (1% ≤ ER ≤ 10%, *N* = 22). Groups were further divided into the HH group (ER > 10%/PR > 20%, *N* = 201), the HL group (ER > 10%/ER 1% ≤ PR ≤ 20% PR, *N* = 60), the LH group (1% ≤ ER ≤ 10%/PR > 20%, *N* = 5), and the LL group (1% ≤ ER ≤ 10%/1% ≤ PR ≤ 20%, *N* = 17). The LH group was excluded due to its small size, leaving the clinical and prognostic characteristics of 2 large groups and 3 subgroups to be analyzed.

**Results:**

*L* group patients had significantly more stage *N*2 axillary lymph nodes than H group patients (31.8% vs. 9.2%, *P* = 0.007). Age (*P* = 0.011), menopause status (*P* = 0.001), and tumor size (*P* = 0.024) were significantly different in the HL vs. HH and LL groups. Five-year DFS (94.6% vs. 77.0%, *P* < 0.001) and 5-year OS (97.2% vs. 85.8%, *P* = 0.001) rates significantly differed between HH and HL. No significant differences in 5-year DFS (77.0% vs. 81.9%, *P* = 0.564) or 5-year OS (85.8% vs. 87.8%, *P* = 0.729) rates were observed between HL and LL; the OS rates of HL and LL were similar.

**Conclusion:**

In the group of ER+/PR+/HER2-patients, there was no significant prognostic difference between ER-low positive and ER-high positive groups, but low PR expression was significantly associated with a worse prognosis. The role of ER and PR balance in breast cancer progression and individualized treatment requires further investigation.

## 1. Introduction

Breast cancer is hormone-dependent; endocrine therapy in patients with estrogen receptor (ER) and progesterone receptor (PR) positive breast malignancies can reduce the risk for breast cancer recurrence and metastasis [1]. Determining the hormone receptor status has become an indispensable aspect of diagnosing and treating breast cancer and can predict patient prognosis and the possibility of response to endocrine therapy [2] and chemotherapy [3].

Studies have shown that approximately 70% of breast cancers express ER [4]. Because the expression of PR is also regulated by ER, PR is also positive in most ER-positive breast cancers [5]. It is generally believed that the efficacy of endocrine therapy in patients positive for both ER and PR is better than that in patients with single positive and double negative expression [6]. It was previously believed that patients with tumors with ER expression levels ≥1% could derive clinical benefit from endocrine therapy [7], but recent studies have revealed that breast tumors with low ER expression (1% ≤ ER ≤ 10%) may have unique molecular and clinical characteristics compared with tumors with higher ER expression (ER > 10%) [8]. In the 2020 update of the ASCO/CAP guidelines, the expert panel acknowledged the limited data on the benefits of endocrine therapy for patients with 1%–10% ER expression and recommended that this group of patients be documented as “ER-low positive” [9]. In the past, PR was considered an indicator of functional ER activity because PR is the product of ER target genes, and the synthesis of PR protein is regulated by ER [10], but recent evidence shows that the regulatory processes of PR function and target genes are separated from ER, which is an independent motor of cell proliferation in breast cancer [11]. ER or PR status is considered an independent risk factor for having a poor prognosis [12, 13], but in clinical practice, we found that ER and PR double-positive tumors also showed significantly different biological characteristics.

Most of the existing studies have been absorbed in the prognosis of breast tumor patients with a single negative ER or PR status or stratified analysis of the expression of a single receptor [14, 15], but there are few clinical studies on the combined stratification of the expression of ER and PR. In this study, ER (10%) [9] and PR (20%) [16] status were included in 283 ER+/PR+/HER2− cases from January 2010 to October 2016 to explore the unique clinical features and the possible prognosis of HER2-negative breast cancer with different hormone receptor balance and to provide a theoretical basis for the treatment and research of patients with hormone receptor-positive breast cancer.

## 2. Patients and Methods

### 2.1. Clinical Case Inclusion

**Figure 1:**
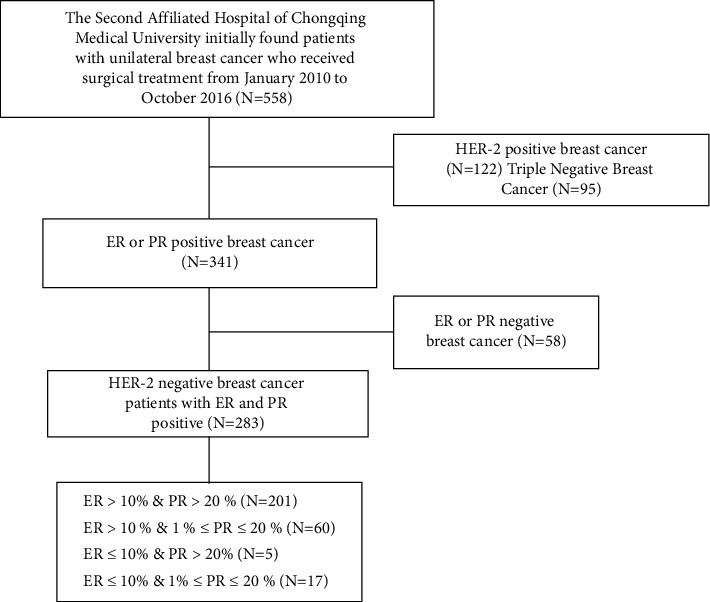
Flow diagram of patients included in this study.

A total of 283 breast cancer patients who met the criteria for ER+/PR+/HER2− breast cancer treated at the Second Affiliated Hospital of Chongqing Medical University from January 2010 to October 2016 were enrolled (Figure 1).

The inclusion criteria of our study were as follows: (1) primary breast cancer; (2) immunohistochemistry showed ER, PR positive, and HER2-negative status; endocrine drugs were taken according to NCCN guidelines after surgery; (3) all patients with positive axillary lymph nodes (ALNs) received chemotherapy with anthracycline plus taxa combined with cyclophosphamide before surgery; (4) sentinel lymph node biopsy was performed before surgery, and ALNs dissection was performed only if metastasis was present; (5) patients who underwent breast-conserving surgery and those patients with positive ALNs received standard radiotherapy according to NCCN guidance; (6) complete clinical records and pathological data were available.

The exclusion criteria of our study were as follows: (1) patients with distant metastasis at onset; (2) systemic malignant tumors other than breast cancer; (3) patients with bilateral primary breast cancer. The patients were first divided into two groups based on ER-low positivity [9]: the high ER expression group (ER > 10%, *H* group, *N* = 261) and the low ER expression group (1% ≤ ER ≤ 10%, *L* group, *N* = 22). According to the criteria of PR20% [16], the patients were further divided into 4 subgroups as follows: the high ER/PR expression group (HH group, ER > 10%/PR > 20%, *N* = 201), the high ER expression/low PR expression group (HL group, ER > 10%/ER 1% ≤ PR ≤ 20% PR, *N* = 60), the low ER expression/high PR expression group (LH group, 1% ≤ ER ≤ 10%/PR > 20%, *N* = 5), and the low ER/PR expression group (LL group, 1% ≤ ER ≤ 10%/1% ≤ PR ≤ 20%, *N* = 17). The sample size of patients in the LH group was too small to be included in the analysis; thus, the clinical and prognostic characteristics of 2 large groups and 3 subgroups were analyzed.

### 2.2. Data Collection and Follow-Up

For the 283 patients included in our study, the clinical data (including age, menopause, pathological type, tumor size, the total number of postoperative axillary lymph node metastases, and mode of operation) were collected by consulting medical records, telephone, and other follow-up methods. The pathological types were divided into two categories for comparison: the first category was noninvasive breast carcinoma (NI) and invasive breast carcinoma of a special type (IST) with better pathological prognosis (intraductal carcinoma, mucinous carcinoma, and tubular carcinoma), and the second category was invasive breast carcinoma of no special type (INST) with relatively poor pathological prognosis (invasive ductal carcinoma and invasive lobular carcinoma). Because premenopausal patients received endocrine therapy with tamoxifen after surgery, postmenopausal patients were treated with aromatase inhibitor endocrine therapy, and they all took medicine regularly; thus, no separate comparison of the efficacy of endocrine drugs was conducted.

All cases received at least one follow-up. The final follow-up date was the date of the last follow-up for deceased patients and those lost to follow-up. The deadline for the final follow-up was October 31, 2021. Where possible, the patients were reexamined every 3 months for 2 years after onset, every half a year between 3 and 5 years, and once a year after 5 years, according to routine advice. Disease-free survival (DFS) was calculated as the interval from radical treatment to tumor local or regional recurrence, distant metastasis, or death due to various causes [17]. Local or regional recurrence refers to the recurrence of the ipsilateral breast, chest wall, or in regional lymph nodes on imaging or pathology. Distant metastasis refers to metastasis that occurred for the first time after surgery and was determined by clinical and imaging examination. Overall survival (OS) refers to the period of any cause between diagnosis and death.

### 2.3. Procedures to Examine ER, PR, HER2, and Ki67

Specimens were fixed in formalin within 30 minutes after harvesting and sent to the Pathology Laboratory of the Second Affiliated Hospital of Chongqing Medical University. The submitted specimens were rinsed with phosphate buffered saline (pH 7.4), fixed, dehydrated step by step with alcohol, embedded in paraffin, cut into 4 *μ*m paraffin sections with a microtome, baked for 2 hours, and stained with hematoxylin-eosin for microscopic observation to confirm the diagnosis of breast malignancies. Immunohistochemistry (IHC) was performed by the SP method. The IHC status of ER, PR, and HER2 was determined by the rabbit monoclonal antibody (clone SP1, SP2, and EP3), and Ki-67 was determined by the mouse monoclonal antibody (clone MX006).

According to ASCO/CAP guidelines, ER, PR, and Ki-67 were localized in cancer cell nuclei, and positive expression was seen as yellowish, brownish yellow, or tan granules in the nuclei. ER and PR positivity criteria of at least 1% of tumor cells showed clear nuclear staining. The Ki-67 score was defined as the percentage of positively stained cells out of the total number of malignant cells. HER2 positivity criteria were IHC 3+ (>30% of invasive tumor cells with homogeneous strong membrane staining) or FISH amplification of the HER2 gene. HER2 negativity was defined as IHC 1+ (faint or barely perceptible incomplete membrane staining in >10% of invasive tumor cells); IHC 0 (no staining observed or incomplete or barely perceptible membrane staining in ≤10% of invasive tumor cells); no over-expression of the HER2 gene in FISH testing. Two independent observers assessed all samples, and any disagreements were agreed upon following a joint review using multiple microscopes.

### 2.4. Statistical Analysis

The collected data were collated and analyzed with IBM SPSS software version 26.0, and the numerical data were analyzed using the chi-square test (or the Fischer exact probability method, if necessary). The survival time was calculated monthly, the survival rate was calculated by the Kaplan–Meier method, then we graphed the survival curve, and the differences in the survival curves were compared by a log-rank test. A Cox proportional hazard model was applied to the univariate and multivariate analysis of clinical and pathological indexes. The level of significance was set at *P* < 0.05.

## 3. Results

### 3.1. Patient Demographics and Baseline Characteristics

Table 1 lists the clinical and demographic characteristics of participants. The median age of the selected patients was 50 years (range 30–87 years), and 47.8% of patients were premenopausal. The ALNs' metastasis status, tumor size, and histological type were mainly T1 (147, 51.9%), *N*0 (188, 66.4%), and INST (241, 86.9%), with Ki-67 < 14% proportion (61.1%, 170). The surgical method was primarily modified radical mastectomy (211, 75.9%). The comparison between the two groups showed that the proportion of axillary lymph nodes with stage *N*2 and above in group *L* was higher than the proportion in group H (31.8% vs. 9.2%, *P* = 0.007), but there was no meaningful difference in the distribution of age (*P* = 0.315), menopause status (*P* = 0.353), pathological type (*P* = 0.430), Ki-67 expression (*P* = 0.321), tumor size (*P* = 0.923), or surgical methods (*P* = 0.876) (*P* > 0.05). The two groups were further divided into four groups according to PR expression levels: HH group (201, 71.0%), HL group (60, 21.2%), LH group (5, 1.8%), and LL group (17, 6.0%). The LH group (5, 1.8%) was not included in the statistical analysis due to its clinical rarity and the small number of included cases. The other three groups were compared using the Fischer exact test, and the comparison between the three groups is shown in Table 2. It was observed that the proportion of patients ≥50 years old in the HL group was 70.0%, which was significantly different from the HH group (48.3%) and LL group (52.9%) (*P* = 0.011); the proportion of postmenopausal patients in the HL group was 73.3%, which was different from 45.8% in the HH group and 52.9% in the LL group (*P* = 0.001); the proportion of tumors T3 and above in the HL group was 8.3%, which was significantly different from the 2.5% in the HH group and 0% in the LL group (*P* = 0.024). Nevertheless, there was no obvious difference in the pathological type (*P* = 0.254), Ki-67 expression (*P* = 0.321), or surgical methods (*P* = 0.882) among the three groups (*P* > 0.05), and clinical characteristics and pathological characteristics did not differ between the HH and LL groups (*P* > 0.05).

### 3.2. Follow-Up and Survival Analysis

**Figure 2:**
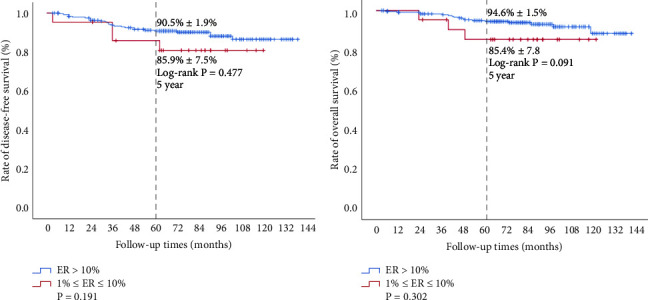
Survival analysis of the high ER (ER > 10%, *H* group) and low ER (1% < ER < 10%, *L* group) group. Kaplan–Meier survival curves were plotted, and the log-rank test was performed to compare the overall survival and disease-free survival between the *H* group and the *L* group.

**Figure 3:**
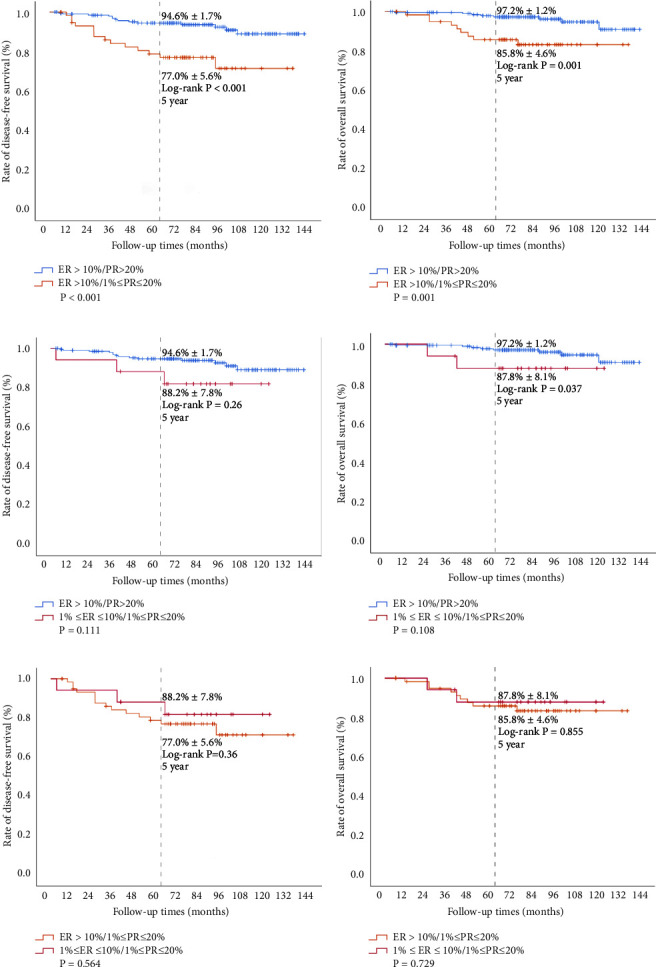
Survival analysis of the HH group (ER > 10%/PR > 20%), HL group (ER > 10%/1% ≤ PR ≤ 20%), and LL group (1% ≤ ER ≤ 10%/1% ≤ PR ≤ 20%). Kaplan–Meier survival curves were plotted, and the log rank test was performed to compare the overall survival and disease free survival among the three groups.

Follow-up data were available for all 283 patients. Because the enrolled patients with ER-positive and PR-positive had relatively good prognoses, the median DFS (survival time corresponding to a 50% cumulative recurrence rate) and median OS (survival time corresponding to a 50% cumulative survival rate) rates were not reached in this study. Although there was no apparent difference in 5-year DFS (90.5% ± 1.9% vs. 85.9% ± 7.5%, *P* = 0.477, Figure 2(a)) and 5-year OS (94.6% ± 1.5% vs. 85.4% ± 7.8%, *P* = 0.091, Figure 2(b)) rates in the HL group, after dividing the subgroups, the 5-year DFS in the HH group (94.6% ± 1.7%) was different from the HL group (77.0% ± 5.6%) (*P* < 0.001, Figure 3(a)), and the 5 year OS in the HH group (97.2% ± 1.2%) also showed a significant difference from that in the HL group (85.8% ± 4.6%) (*P* = 0.001, Figure 3(b)). Although the 5-year DFS rate in the HH group (94.6% ± 1.7%) was not obviously different from the LL group (88.2% ± 7.8%) (*P* = 0.26, Figure 3(c)), the 5-year OS rate in the HH group (97.2% ± 1.2%) showed a significant difference compared with that in the LL group (87.8% ± 8.1%) (*P* = 0.037, Figure 3(d)). There were no obvious differences in 5-year DFS (77.0% ± 5.6% vs. 88.2% ± 7.8%, *P* = 0.36, Figure 3(e)) or 5-year OS (85.8% ± 4.6% vs. 87.8% ± 8.1%, *P* = 0.855, Figure 3(f)) rates between the HL and LL groups, and the OS of the HL and LL groups tended to be consistent. Overall, ER low positivity may not represent a poor prognosis in terms of OS and DFS rates in the patient population with both ER and PR positivity, but low PR expression is associated with significantly worse DFS and OS rates.

The clinical and pathological characteristics and surgical methods of 278 patients were introduced into the multivariate regression analysis which was based on the Cox proportional hazards model (Table 3). The results showed that for ER+/PR+/HER2− patients, the risk for recurrence of T3 tumors was 5.862 times that of T1 tumors (95% CI 1.392–24.68; *P* = 0.016); the risk for postoperative recurrence was 3.278 higher for *N*1 axillary lymph nodes than *N*0 (95% CI 1.12–9.591; *P* = 0.003); the risk for postoperative recurrence was 10.914 higher for *N*2 axillary lymph nodes than *N*0 (95% CI 4.073–29.24; *P* < 0.001); when ER expression was >10%, the risk for recurrence of 1% ≤ PR ≤ 20% tumors was 2.87 times that of PR > 20% (95% CI 1.196–6.887; *P* = 0.018) tumors, and the differences were statistically significant (*P* < 0.05). However, age, menopausal status, pathological type, Ki-67 status, and surgical approach did not affect the DFS rate, and the difference did not seem to be significant (*P* > 0.05).

The risk of death among postmenopausal patients was 8.466 times that among premenopausal patients (95% CI 1.571–45.618); *P* = 0.013); the risk of death in postoperative axillary lymph node *N*1 patients was 5.053 times that in *N*0 patients (95% CI 1.313–19.444; *P* = 0.018); the risk of death in postoperative axillary lymph node *N*2 patients and above was 7.594 times that in N0 patients (95% CI 1.897–25.949; *P* = 0.004); when ER > 10%, the risk of death in 1% ≤ PR ≤ 20% patients was 3.813 times that in PR > 20% patients (95% CI 1.204–12.077; *P* = 0.023). However, age, pathological type, Ki-67 status, and surgical approach did not affect the OS rate, without being obviously significant (*P* > 0.05).

The HL group had a 2.87-fold greater risk for relapse (95% CI 1.196–6.887; *P* = 0.018) and a 3.813-fold greater risk of death (95% CI 1.204–12.077; *P* = 0.023) than the HH group. There was no obvious difference in the risk for recurrence (*P* = 0.339) or death (*P* = 0.111) between the LL group and the HH group due to the small number of LL group patients; the risk for recurrence (*P* = 0.574) and death (*P* = 0.981) was not obviously different in the HL group vs. the LL group. In the multivariate regression analysis of 283 patients, the risk for recurrence in the *L* group did not differ from the H group (HR = 1.065, 95% CI 0.318–3.566; *P* = 0.918), and the mortality risk in the *L* group was 2.606 times than the H group (95% CI 0.685–9.913); nevertheless, the differences were not significant at the statistical analysis (*P* = 0.160) and does not indicate that ER-low positivity is an independent risk factor of OS rates in the ER+/PR+/HER2 breast cancer patient populations studied.

## 4. Discussion

Breast cancer is highly heterogeneous, especially in patients with hormone receptor positivity, and there are substantial differences in biological behaviors, treatment responses, and prognoses among patients [18]. Breast cancer with ER expression from 1% to 10% is a clinically and biologically heterogeneous disease. It is known that hypermethylation of the ER promoter [19], histone acetylation and microRNAs [20], and inhibition of MAPK signaling pathways [21] may lead to loss and re-expression of ER, resulting in low expression or no expression of ER. Some researchers believe that the ER immunophenotype results from inadequate tissue fixation or immunohistochemical failure [22]. However, a blooming number of studies have shown that the ER-/PR+ phenotype is real, is significantly correlated with younger age and higher histological grade [23], and is an independent risk factor for bad prognosis [12]. Low ER expression has thus become a research focus. Because of the low incidence of this status and the limited evidence, the clinical characteristics and treatment recommendations did not appear in the latest guidelines, and there remain many challenges to be explored.

In our study, the proportion of *N*2 and above axillary lymph nodes in the *L* group was larger than the *H* group (31.8% vs. 9.2%), but there was no obvious difference in age distribution, menopause status, pathological type, Ki-67 expression, tumor size, or mode of surgery. The results of a previous retrospective study showed that there were differences in age, clinical stage, ductal carcinoma, and preoperative chemotherapy response between patients with ER-low positive and those with high expression of ER [8]. These results appear to contradict our findings; however, the earlier study also included PR-negative patients, and earlier studies have demonstrated that PR-negative status is an independent driver for poor prognosis [24], which may be one of the reasons for this difference.

According to reports, the annual recurrence pattern of ER-low positive breast cancer patients is similar to that of triple-negative or ER-negative patients. That is, five years after the onset of the disease, the annual recurrence rate was higher (1.5%–3.5%), and 5 to 10 years after diagnosis, the annual recurrence rate dropped to 1%–3%. Patients with high ER-positive tumors had a low annual recurrence rate of 5 years after the onset of the disease (1.0%–2.5%), but 5 to 10 years after diagnosis (2.5%–4.0%), the annual recurrence rate increased exponentially and was higher than in ER low positive patients [25]. This was also reflected in the results of our study. Although there were no obvious differences in OS and DFS rates, there were still obvious differences in 5-year DFS (90.5 ± 1.9% vs. 85.9 ± 7.5%) and 5-year OS (94.6 ± 1.5% vs. 85.4% ± 7.8%) rates between the *H* group and the *L* group, and the survival curves of the two groups were similar after 5 years. Some researchers have suggested that there is no significant interaction between ER percentage staining or ER intensity, endocrine therapy, and breast cancer-specific survival rates using multivariate models [26]. Other studies have shown that when the expression of ER is 1% to 9%, tamoxifen does not affect prognosis. However, when the expression of ER was high (ER ≥ 10%), 5-year tamoxifen treatment could significantly improve patient prognosis [27]. However, patients who were PR and HER2 positive were not excluded from the above studies. In our study, after excluding the above factors, the ER-low positive group had similar DFS and OS rates to the high ER expression group, which may suggest that the effect of endocrine therapy in the group with ER low positivity over a longer period may need to be reevaluated to avoid poor prognoses resulting from a lack of endocrine therapy.

Some studies have revealed that the prognosis and predictive value of PR mainly depend on the expression of PR and the activity of ER, and the absence of PR expression resulted in the loss of ER function and improved tolerance of endocrine therapy [28]. Related studies suggested that compared with ER+/PR+ tumors, ER+/PR− tumors express higher levels of the tumor growth factor signaling pathway genes, and growth factors can reduce the PR expression level of ER+/PR− tumors, enhancing resistance to endocrine therapy [24]. Nevertheless, a meta-analysis of patients with early breast cancer has shown that in ER-positive diseases, PR status or level is not related to recurrence and mortality [1], and similar data were observed in another smaller randomized controlled study [6]. Some studies have suggested that PR expression greater than 20% can be used as an empirical cutoff value to predict the difference in the survival rate between luminal A and luminal B tumors [16]. The Chinese Society of Clinical Oncology (CSCO) guidelines also recommend PR20% positivity as the critical determinant of luminal A vs. luminal B tumors [29]. Therefore, to observe the effect of ER expression on prognosis in different PR states, we further divided the patients into four subgroups according to PR ≤ 20% or PR > 20% status. The results demonstrated that besides the HH group and LL group, the HL group had a larger proportion of patients aged 50 and over (70%, *P* = 0.011), more menopausal patients (73.3%, *P* = 0.001), and a larger proportion of T3 and above tumors (8.3%, *P* = 0.024), which is consistent with previous reports regarding the low expression of PR [16]. Although there was no obvious difference between the HL group 5-year OS (85.8% ± 4.6%) and LL group 5-year OS (87.8% ± 8.1%) rates, the 5-year OS rates of both groups were obviously worse than the HH group (97.2 ± 1.2%). Early studies also showed that PR levels were significantly correlated with DFS and OS rates of patients with ER-positive metastatic diseases who received adjuvant therapy [30]. In the subgroup analysis, we also found an obvious difference in the risk for recurrence and death between the HH group and the HL group, showing that the risk for recurrence in the HL group was 2.87 times greater than the HH group (95% CI 1.196–6.887; *P* = 0.018), and the mortality risk was 3.813 times greater than the HH group (95% CI 1.204–12.077; *P* = 0.023), indicating that the imbalance of ER and PR expression had a greater impact on DFS and OS rates in ER+/PR+/HER2− patients. The LH group was not included in the statistical analysis because of its small sample size. In this group, only one patient experienced recurrence in the third year after surgery and died in the fourth year. None of the other LH group patients experienced recurrence or metastasis. This subgroup is very rare in clinical practice, and there are few previous studies and reports available. Our team will focus on the clinical characteristics and prognoses of this subgroup in the future. Some endocrine therapy research studies have illustrated that compared with the expression of ER, the expression of PR can better predict the therapeutic response to tamoxifen [10]; that is, the efficacy of tamoxifen is worse when the expression of PR is low. Some studies have also revealed that there is no marked difference in the response of luminal A and B tumors to aromatase inhibitors [31], which may suggest that although the risk for recurrence in the low PR expression group is relatively high, excessive drug use should be avoided during endocrine therapy to limit side effects, while the improvement in the prognosis of patients may not be significant.

The sample size of our study was relatively small, and more research is required to support the results. The limitations of our study also include the lack of unified surgical treatment because of the requirement for modified radical mastectomy for some patients who met the indications for breast conservation, although researchers have indicated that the DFS rate of breast-conserving surgery combined with radiotherapy is not significantly different from modified radical mastectomy in Chinese patients with primary breast cancer [32]. We also analyzed the surgical treatment of the patients and found that it did not have a significant impact on prognosis. All the patients were treated with standard endocrine drugs after surgery; thus, it was not possible to compare the prognostic impact of endocrine therapy. These are the shortcomings of this study but also the directions for further research.

## 5. Conclusion

Our study revealed that although previous research studies have suggested that the prognosis of ER-low positive patients is better than ER-high positive patients, there was no obvious difference between the two in the groups of ER+/PR+/HER2− patients, while when PR was weakly positive, some patients had a poor prognosis even if the ER expression was high. In the population with ER-low positive patients, some patients were PR negative, after excluding PR negative patients, and when PR-positive and HER2-negative patients were excluded, the prognosis of ER-low positive patients was slightly worse than that of ER-high positive patients; however, the difference was not considered statistically significant. This suggests that poor prognosis in the ER-low positive population is also mainly related to PR negativity. However, this hypothesis needs to be further explored. We cannot simply regard PR as a marker of functional ER, and further distinction of the role of PR subtypes in the early and advanced stages of breast cancer progression to guide treatment is needed. Therefore, ER-low positive and PR-low positive patients require further study in terms of clinical and molecular mechanisms and their relation to treatment strategies.

## Figures and Tables

**Table 1:** Tumor and patient characteristics stratified by the ER statue.

Characteristics	Total *N* = 283 (%)	ER >10% *N* = 261 (%)	1% ≤ ER < 10% *N* = 22 (%)	*P* value
Age (y)				0.315
<50	134 (47.3%)	122 (46.7%)	12 (54.5%)	
≥50	149 (52.7%)	139 (53.3%)	10 (45.5%)	
Menstrual status				0.353
Pre/Peri-	137 (48.4%)	125 (47.9%)	12 (54.5%)	
Post-	146 (51.6%)	136 (52.1%)	10 (45.5%)	
Histology type				0.430
NI＆IST	37 (13.1%)	35 (13.4%)	2 (9.1%)	
NST	246 (86.9%)	261 (86.6%)	20 (90.9%)	
Ki-67				0.321
<14%	173 (61.1%)	158 (60.5%)	15 (68.2%)	
≥14%	110 (38.9%)	103 (39.5%)	7 (31.8%)	
Tumor size (cm)				0.923
T1	147 (51.9%)	136 (52.1%)	11 (50.0%)	
T2	126 (44.5%)	115 (41.1%)	11 (50.0%)	
T3	8 (2.8%)	8 (3.1%)	0 (0.0%)	
T4	2 (0.7%)	2 (0.8%)	0 (0.0%)	
ALN status				**0.007**
0	188 (66.4%)	175 (67.0%)	13 (59.1%)	
1–3	64 (22.6)	62 (23.8%)	2 (9.1%)	
≥4	31 (11.0%)	24 (9.2%)	7 (31.8%)	
Stage				**0.011**
0–I	114 (40.3%)	105 (40.2%)	9 (40.9%)	
II	133 (47.0%)	127 (48.7%)	6 (27.3%)	
III	36 (12.7%)	29 (11.1%)	7 (31.8%)	
Surgical procedure				0.876
BCS	27 (9.6%)	25 (9.6%)	2 (9.5%)	
SM	16 (5.7%)	16 (6.1%)	0 (0.0%)	
MRM	214 (75.9%)	197 (75.5%)	17 (81.0%)	
ERM	25 (8.9%)	23 (8.8)%	2 (9.5%)	

(NI: noninvasive breast carcinoma; IST: invasive breast carcinoma of special type; INST: invasive breast carcinoma of no special type; BCS: breast-conserving surgery; SM: simple mastectomy; MRM: modified radical mastectomy; ERM: extended radical mastectomy).

**Table 2:** Tumor and patient characteristics stratified by ER and PR statue.

Characteristics	Total *N* = 278 (%)	ER > 10% & PR>20% *N* = 201 (%)	ER > 10%&1% ≤ PR ≤ 20% *N* = 60 (%)	1% ≤ ER < 10%&1% ≤ PR ≤ 20% *N* = 17 (%)	*P* value
Age (y)					**0.011**
<50	130 (46.8%)	104 (51.7%)	18 (30.0%)	8 (47.1%)	
≥50	148 (53.2%)	97 (48.3%)	42 (70.0%)	9 (52.9%)	
Menstrual status					**0.001**
Pre/Peri-	133 (47.8%)	109 (54.2%)	16 (26.7%)	8 (47.1%)	
Post-	145 (52.2%)	92 (45.8%)	44 (73.3%)	9 (52.9%)	
Histology type					0.254
NI&IST	37 (13.3%)	23 (11.4%)	12 (20.0%)	2 (11.8%)	
INST	241 (86.7%)	178 (88.6%)	48 (80.0%)	15 (88.2%)	
Ki-67					0.308
<14%	170 (61.2%)	126 (62.7%)	32 (53.3%)	12 (70.6%)	
≥14%	108 (38.8%)	75 (37.3%)	28 (46.7%)	5 (29.4%)	
Tumor size (cm)					**0.024**
T1	147 (52.2%)	108 (53.7%)	28 (46.7%)	9 (52.9%)	
T2	123 (44.2%)	88 (43.8%)	27 (45.0%)	8 (47.1%)	
T3	8 (2.9%)	5 (2.5%)	3 (5.0)	0 (0.0%)	
T4	2 (0.7%)	0 (0.0%)	2 (3.3%)	0 (0.0%)	
ALN status					0.190
0	186 (66.9%)	137 (68.2%)	38 (63.3%)	11 (64.7%)	
1–3	64 (23.0%)	51 (25.4%)	11 (18.3%)	2 (11.8%)	
≥4	28 (10.1%)	13 (6.5%)	11 (18.3%)	4 (23.5%)	
Stage					**0.008**
0–I	112 (40.3%)	83 (41.3%)	22 (36.7%)	7 (41.2%)	
II	133 (47.8%)	103 (51.2%)	24 (40.0%)	6 (35.3%)	
III	33 (11.9%)	15 (7.5%)	14 (23.3%)	4 (23.5%)	
Surgical procedure					0.882
BCS	27 (9.7%)	18 (9.0%)	7 (11.7%)	2 (11.8%)	
SM	16 (5.8%)	11 (5.5%)	5 (8.3%)	0 (0.0%)	
MRM	211 (75.9%)	153 (76.1%)	44 (73.3%)	14 (82.4%)	
ERM	24 (8.6%)	19 (9.5)%	4 (6.7%)	1 (5.9%)	

**Table 3:** Multivariate analysis of factors associated with DFS and OS.

Characteristics	DFS		OS	
	HR (95% CI)	*P* value	HR (95% CI)	*P* value
Age (y; ≥50 vs.<50)	0.44 (0.148–1.307)	0.139	0.537 (0.136–2.118)	0.375
Menstrual status (post-vs. Pre-)	3.015 (0.971–9.359)	0.056	8.466 (1.571–45.618)	**0.013**
Histology type (NST vs. NI&IST)	1.196 (0.287–4.978)	0.805	0.841 (0.189–3.741)	0.82
Ki-67 (≥14% vs. <14%)	1.013 (0.457–2.246)	0.974	0.695 (0.218–2.216)	0.538
Tumor size (cm)		**0.039**		**0.015**
T2 vs. T1	0.904 (0.39–2.096)	0.814	0.385 (0.119–1.252)	0.113
T3 vs. T1	5.862 (1.392–24.68)	**0.016**	4.421 (0.823–23.758)	0.083
T4 vs. T1	2.455 (0.367–16.42)	0.354	5.719 (0.45–72.68)	0.179
ALN status		≤**0.001**		**0.008**
1–3 vs. 0	3.278 (1.12–9.591)	**0.003**	5.053 (1.313–19.444)	**0.018**
≥4 vs. 0	10.914 (4.073–29.24)	≤**0.001**	7.594 (1.897–25.949)	**0.004**
Breast surgery		0.377		0.104
SM vs. BCS	0.405 (0.035–4.726)	0.471	0.493 (0.041–5.99)	0.579
MRM vs. BCS	0.333 (0.091–1.224)	0.098	0.184 (0.043–0.785)	**0.022**
ERM vs. BCS	0.526 (0.098–2.83)	0.454	0.415 (0.06–2.879)	0.373
Group		0.061		0.052
ER > 10%&1% ≤ PR ≤ 20% vs. ER > 10% &PR>20%	2.87 (1.196–6.887)	**0.018**	3.813 (1.204–12.077)	**0.023**
1% ≤ ER ≤ 10%&1% ≤ PR ≤ 20% vs. ER > 10% &PR > 20%	1.963 (0.492–7.83)	0.339	3.738 (0.738–18.936)	0.111
1% ≤ ER ≤ 10%&1% ≤ PR ≤ 20% vs. ER > 10%&1% ≤ PR ≤ 20%	0.684 (0.182–2.573)	0.574	0.98 (0.192–5.014)	0.981

## Data Availability

The data that support our findings of this study are available from the corresponding author upon reasonable request.
